# Fear of Falls & Quality of Life in Community-Dwelling Older Adults: Is Diabetes an Effect Modifier?

**DOI:** 10.1093/geroni/igaa057.3333

**Published:** 2020-12-16

**Authors:** Christina Whitehouse, Janell Mensinger, Michelle McKay

**Affiliations:** Villanova University, Villanova, Pennsylvania, United States

## Abstract

Prior research suggests that diabetes is a risk factor for falling. Moreover, older adults with diabetes are more likely to experience hip fractures when compared to older adults without diabetes. Research has also shown a relationship between fear of falling and quality of life. This study aimed to examine the moderating role of diabetes in the relationship between fear of falling (FoF) and quality of life (QoL) among older adults in a program for all-inclusive care for the elderly (PACE). This was a retrospective single cohort study that included 84 older adults in a PACE program located in the Northeastern United States. Participants were 55 years of age or older (M=70.33; SD=6.46) and cognitively intact. Diabetes diagnosis (n=46) was determined according to chart review. Fear of Falling was assessed with the Falls Efficacy Scale-International, and the Short Form 12v2 (SF-12v2) was used to measure the quality of life, including physical and mental domains. Hayes’ Process Macro was used to conduct moderation analyses (model 1) controlling race, gender, age, and comorbidities. Alpha was set at .10 to account for low power to detect interaction effects with small groups. Our results indicate the interaction between diabetes status and FoF was significant for mental QoL (p=.09) and not significant for physical QoL (p=.99). The association between FoF with lower mental QoL was stronger for patients with diabetes than for patients without diabetes; this finding was not replicated for physical QoL. Regardless of diabetes status, physical QoL significantly decreased as FoF increased.

